# Obesity in Scotland: a persistent inequality

**DOI:** 10.1186/s12939-017-0599-6

**Published:** 2017-07-27

**Authors:** Elaine Tod, Catherine Bromley, Andrew D. Millard, Allan Boyd, Phil Mackie, Gerry McCartney

**Affiliations:** 1 Public Health Observatory, NHS Health Scotland, Meridian Court, 5 Cadogan Street, Glasgow, G2 6QE Scotland UK; 2 Public Health Observatory, NHS Health Scotland, Gyle Square, Edinburgh, EH12 9EB Scotland UK; 3 NHS Greater Glasgow and Clyde, 1 Smithills Street, Paisley, PA1 1EB Scotland UK; 4 Scottish Public Health Network, NHS Health Scotland, Meridian Court, 5 Cadogan Street, Glasgow, G2 6QE Scotland UK

**Keywords:** Inequality, Obesity, Overweight, BMI, Scotland, Scottish Health Survey, Adults, Children, Weight, Trends

## Abstract

**Background:**

Obesity is a health problem in its own right and a risk factor for other conditions such as cardiovascular disease. The prevalence of overweight and obesity increased in Scotland between 1995 and 2008 with socio-economic inequalities persisting in adults over time and increasing in children. This paper explores changes in the underlying distribution of body mass index (BMI) which is less well understood.

**Methods:**

Using data from the Scottish Health Survey (SHeS) between 1995 and 2014 for adults aged 18–64 years, we calculated population distributions for BMI for the population overall, and for age, sex and deprivation strata. We used SHeS data for children aged 2–15 years between 1998 and 2014, in addition to data from the Child Health Systems Programme (CHSP) collected from primary one (P1) children in participating local authorities, to describe the overall trends and to compare trends in inequalities by deprivation strata.

**Results:**

Amongst adults, the BMI distribution shifted upwards, with a large proportion of the population gaining a small amount of weight between 1995 and 2008 before subsequently stabilising across the distribution. In men the prevalence of obesity showed a linear deprivation gradient in 1995 but over time obesity declined in the least deprived quintile while the remaining four quintiles converged (and stabilised). In contrast, a persistent and generally linear gradient is evident among women for most of the 1995–2014 period.

For those aged 2–15 years, obesity increased between 1998 and 2014 for the most deprived 40% of children contrasted with stable trends for the least deprived. The surveillance data for P1 children in Scotland showed a persistent inequality between 2005/06 and 2014/15 though it was less clear if this is widening.

**Conclusions:**

The BMI distribution for adults increased between 1995 and 2008 with a large proportion of the population gaining a small amount of weight before stabilising across the distribution. Inequalities in obesity persist for adults (with different underlying patterns evident for men and women), and may be widening for children. Actions to reduce the obesogenic environment, including structural changes not dependent on individual agency, are urgently needed if the long-term health, social and inequality consequences of obesity are to be reduced.

**Electronic supplementary material:**

The online version of this article (doi:10.1186/s12939-017-0599-6) contains supplementary material, which is available to authorized users.

## Background

An increase in the prevalence of obesity is in many ways an inevitable consequence of living in a society where relatively cheap, energy dense foods are marketed relentlessly and where physical activity becomes dissociated from the normal means of getting around and working [1, 2]. Obesity represents a health problem in its own right, but also a risk for other associated health problems such as heart disease, osteoarthritis and cancer. As such it contributes to a substantial burden of disease in the population [3]. Although the prevalence of obesity has increased across the globe in recent years, there is now some evidence that it is stabilising internationally [4]. However, while population-levels of obesity can stabilise, this can mask ongoing increases at the upper end of the distribution [5]. Socio-economic inequalities in obesity in Scotland have been shown to have persisted in adults over time, while increasing in children [6]. Within specific Scottish cohorts, socio-economic inequalities in obesity have also been noted [7]. However, these patterns have not yet been fully explored and do not form part of the Scottish Government’s routine inequalities monitoring framework [8]. This paper seeks to explore the trends in Body Mass Index (BMI) distribution and prevalence of obesity by area deprivation over time for adults and children to clarify the extent and nature of obesity inequalities in Scotland.

## Methods

### Data sources

Data were analysed from ten waves (1995, 1998, 2003, 2008, 2009, 2010, 2011, 2012, 2013 and 2014) of the Scottish Health Survey (SHeS) for adults, with child data included from 1998 onwards. The SHeS is described in more detail elsewhere [9, 10]. Briefly, the SHeS is a stratified cluster probability survey of the non-institutionalised population resident in Scotland. The survey was conducted intermittently prior to 2008 after which the survey design changed to provide annual data but with a smaller sample size each year. The age range included also changed over time with adults aged 16–64 years, 16–74 years and >16 years included in 1995, 1998 and from 2003 onwards respectively (although we used only the data for adults aged 18–64 years to achieve consistency over time). Children aged 2–15 years were included from 1998 onwards (with 0–1 year olds included from 2003, but BMI data has not been collected for this age group). Interviewers collected a range of data on the social circumstances and health behaviours of the population alongside a range of anthropometric and biological measures. Relevant to this study, the interviewers objectively measured the (minimally clothed) height and weight of respondents (aged >2 years) which allowed the calculation of BMI (weight in kilograms divided by the square of height in metres). The BMI data were stratified by age, sex and quintiles of the Scottish Index of Multiple Deprivation (SIMD) (based on the household’s postcode). The SIMD identifies small areas of deprivation across Scotland in a consistent way, ranking areas from most to least deprived. It is constructed from seven domains: income, employment, crime, education, health, housing and geographic access to services [11]. SIMD is a relative measure of deprivation, it cannot say how much more deprived one area is compared to another only whether an area is worse off relative to other areas. SIMD was evenly distributed in the survey data with approximately 20% of the population in each quintile.

For children, we also used data from 2005/6 onwards from the Child Health Systems Programme (CHSP) School which collects data from children in their first year of school i.e. primary one (P1) children (aged 4–6 years) in participating schools in Scotland. As part of this programme, objective height and weight measures are taken alongside postcode information which allows mapping to SIMD deprivation quintiles. The participation of schools in the system has increased over time with approximately 48% of P1 children included in 2005/06 rising to 92% by 2014/15 (Table 1) [12]. Privately funded schools (with some exceptions) generally do not participate and there have been unrepresentative samples taken in some local authority areas, particularly nearer the beginning of the time series [13]. These data are not routinely published separately for boys and girls.

**Table 1:** Characteristics of the samples used: Scottish Health Survey (SHeS) for adults and children; Child Health Systems Programme (CHSP) Schools for children

SHeS							CHSP		
	Adults			Children			Children in P1		
Year	Sample size (with valid BMI values) 18–64 yrs	Response rate (height)^b^	Response rate (weight)^b^	Sample size (with valid BMI values) 2–15 yrs	Response rate SHeS (height)^c^	Response rate SHeS (weight)^c^	Year	Sample size	% of P1 children in Scotland included in CHSP
1995	7115	78%	75%	N/A	N/A	N/A	-	-	
1998	6707	73%	71%	3486	72%	71%	-	-	
2003^a^	5092	53%	51%	2425	60%	59%	-	-	
2005	-	-	-	-	-	-	2005/6	25,879	47.6%
2006	-	-	-	-	-	-	2006/7	25,213	47.2%
2007	-	-	-	-	-	-	2007/8	28,284	54.2%
2008	3986	47%	46%	1298	41%	45%	2008/9	34,472	65.4%
2009	4660	49%	47%	1886	45%	47%	2009/10	40,209	73.9%
2010	4447	48%	46%	1236	41%	41%	2010/11	41,213	74.4%
2011	4556	47%	46%	1272	39%	39%	2011/12	52,545	94.2%
2012	2951	49%	48%	1281	46%	46%	2012/13	54,499	95.6%
2013	3094	49%	48%	1311	46%	45%	2013/14	54,944	92.4%
2014	2851	49%	48%	1214	48%	47%	2014/15	54,761	91.6%

^a^The sample design changed in 2003 to include the whole population (0+ years), with all adults within selected households eligible to be interviewed (compared with just one in 1995 and 1998). The decline in response rates is partly attributable to these changes

^b^Height and weight measurements were attempted for all adults. From 2003 onwards these response figures include those >65 years among whom response to measurements is lowest (overall response rates by age group are not available). The analyses presented in this paper are based only on adults aged 18–64 years

^c^Height and weight were measured in children aged 2–15 years but the response rate denominator is based on the 0–15 years age group, so these will be underestimates

### Analysis of data

The trend over time in obesity prevalence by SIMD quintile charts present two-year rolling averages from 2008 onwards for adults, and three-year rolling averages for children, to smooth the increased sampling volatility for population sub-groups at the latter end of the time series. Statistical significance was assessed using binary logistic regression (with obesity as the outcome). We also calculated the Relative Index of inequality (RII) and Slope Index of Inequality (SII) across SIMD quintiles for men and women. The RII measured the gradient in obesity prevalence relative to the mean level in the whole population. The SII is the slope of the “best fit” regression line measuring the relationship between obesity prevalence and SIMD quintile (an SII of 0 would indicate no inequalities, while the higher the SII value the greater the inequality) [14].

The proportion of the Scottish adult population (18–64 years) at each gradation of Body Mass Index (BMI) was calculated and stratified by age, sex and SIMD quintile. For the adult BMI distributions stratified by age and sex we presented smoothed data by taking a mean of each data point with its two neighbours. Quantile regression at the 10^th^, 25^th^, 50^th^, 75th and 90^th^ quantiles was carried out to test for changes in the BMI distribution over time on the un-smoothed data.

For adults, obesity was defined as a BMI of 30 kg/m^2^ or more. To aid the interpretation of the trends, the BMI values at the median, 5th and 95th centiles of the sample distribution were also plotted. For children, the proportion aged 2–15 years at each gradation of body mass was calculated and also smoothed in the same way as for adults. To account for the potential effect of changing age distribution over time (a decrease in older children and an increase in younger children) age-standardised trends (using the 1998 child population distribution) were also graphed. Both curves were very similar indicating that changes in the age distribution had minimal effect on the trends observed. For children, we used the epidemiological classification threshold for obesity risk (>95th centile in comparison to the 1990 UK growth reference standards).

## Results

### Sample characteristics

The characteristics of the sample of individuals we included in our analyses are given in Table 1. The response rate for SHeS declined over time, as did the sample size. In contrast, the CHSP data for children became more robust over time with the larger sample sizes reflecting increased participation by schools in the programme.

### Trends in the population BMI distribution

**Fig. 1:**
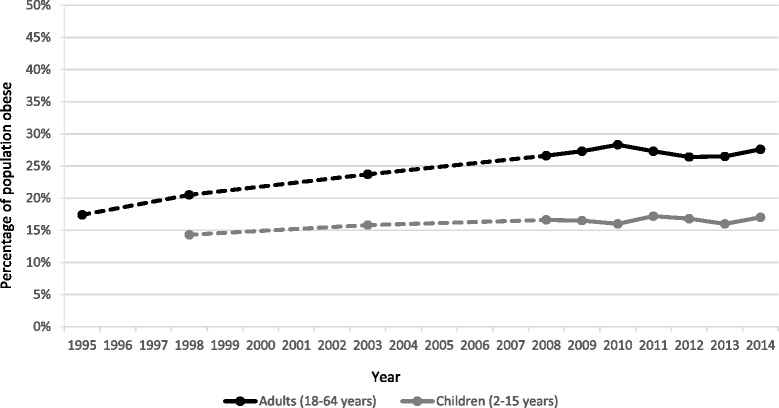
Trends in the percentage of adults and children with a BMI in the obese range. Note: adult obesity = BMI >30; child obesity risk = BMI >95th centile of UK reference charts

Between 1995 and 2008 the percentage of the population aged 18–64 years with a BMI in the obese range increased from 17% (95% CI 16–18%) to 27% (95% CI 25–28%), after which it stabilised at 26–28% (Fig. 1). Similar patterns were seen for men and women (data not shown). In contrast, levels of epidemiologically-defined obesity risk in children aged 2–15 years have been somewhat more stable, ranging non-significantly from 14% (CI 13–16%) to 17% (CI 15–20%) in the 1998–2014 period (Fig. 1). Interpreting trends for P1 children using the CHSP data is difficult due to the changes over time in the number of areas participating in the surveillance programme. However, the data from 2005/6 onwards (the baseline used in official reporting) also suggest that levels of obesity risk have been fairly stable at 9–11% (data not shown).

Mean BMI in adults aged 18–64 years increased from 26.0 kg/m^2^ (CI 25.8–26.1) in 1995 to 27.4 kg/m^2^ (27.2–27.6) in 2008 with no significant variations in the remaining years. Logistic regression confirmed that across the time-period from 1995 to 2014 there was a significant increase in obesity prevalence (p < 0.01) but separate testing showed a lack of significant change from 2009 onwards (p = 0.75). The results of the equivalent tests for the SHeS child data confirm the lack of a significant change in obesity prevalence over time for all children (p = 0.36).

**Fig. 2:**
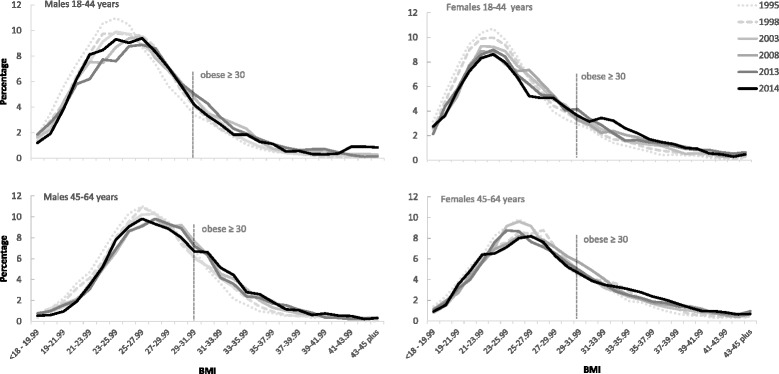
The percentage of males and females aged 18–44 years and 45–64 years at each gradation of BMI from 1995 to 2014. Note: Selected years of data are shown to maintain reasonably consistent time intervals between *lines*. The BMI gradations on the X axis overlap as they are 3-point moving averages. See Additional file 7: Figure S1 for the % of all adults at each gradation of BMI from 1995 to 2014

Figure 2 illustrates the change in the BMI distribution over time by sex and broad age group. All charts show a gradual but consistent upwards shift over time across the entire BMI distribution between 1995 and 2008 with a small shift backwards thereafter (the data for 2009, 2010, 2012 and 2013 have been omitted to provide a more consistent time gap between the survey waves and to make the figure clearer). It can also be seen from Figure 2 that the increase in the prevalence of adult obesity is due to a combination of the entire population distribution moving to the right (i.e. a large proportion of the population having gained a small amount of weight) and to a change in the shape of the distribution (whereby the distribution has become more skewed towards higher BMI values).

The results from the quantile regression analysis confirm that the rate of change in BMI over time was not constant across the BMI distribution for men and women. Additional file 1: Table S1 shows the coefficients for all adults and by sex and broad age group for the 10^th^, 25^th^, 50^th^, 75^th^ and 90^th^ quantiles, compared with the coefficient for the conditional mean from an ordinary least squares regression. See Additional file 2: Figure S2A and Additional file 3: Figure S2B for the distribution of the coefficients at each quantile compared against the coefficient for the mean value of the distribution.

**Fig. 3:**
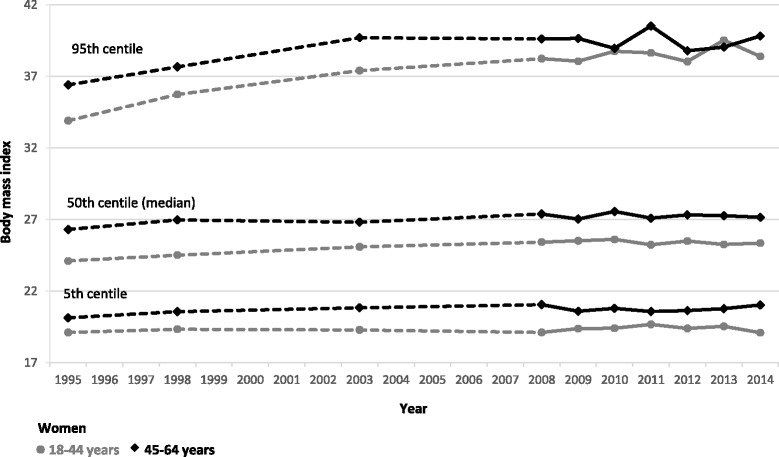
BMI values at the 5th, median and 95th centiles for women, from 1995 to 2014 stratified by age

**Fig. 4:**
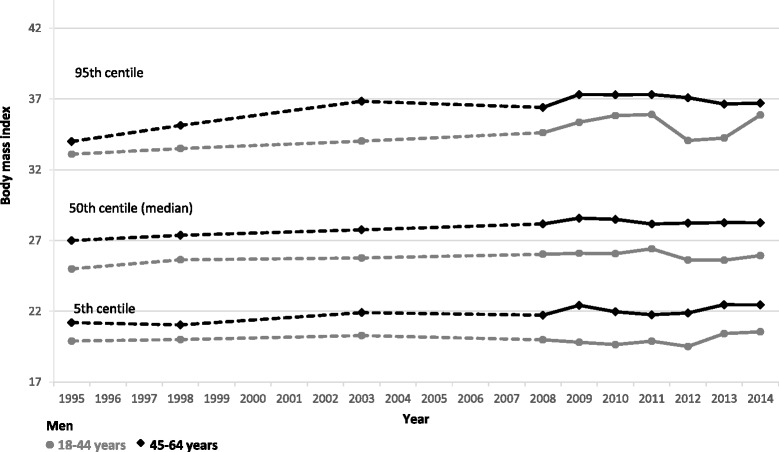
BMI values at the 5th, median and 95th centiles for men, from 1995 to 2014 stratified by age

Figure 3 shows BMI values at the 5^th^, median and 95^th^ centiles for women by age group. The higher prevalence of obesity among women aged 45–64 years, compared with those aged 18–44 years, has been largely constant over time though the gap between the two age-groups appeared to narrow in recent years. In contrast, BMI at the 95^th^ centile has remained largely constant between men in the two age groups over time (Fig. 4). Comparing men with women, BMI values at the 95th centile are consistently higher for women than men in both age groups, though both sexes have seen these values increase over time. In contrast, the gap between men and women’s BMI values at the median and 5th centile is much smaller in all years, with men’s values consistently marginally higher than women’s.

The proportion of adults aged 18–44 years who were obese increased by eight percentage points between 1995 and 2010 (14 to 22%). Adults aged 45–64 years saw a larger equivalent increase in obesity, from 23 to 35% in the same period. Figures in both groups have been stable since then.

**Fig. 5:**
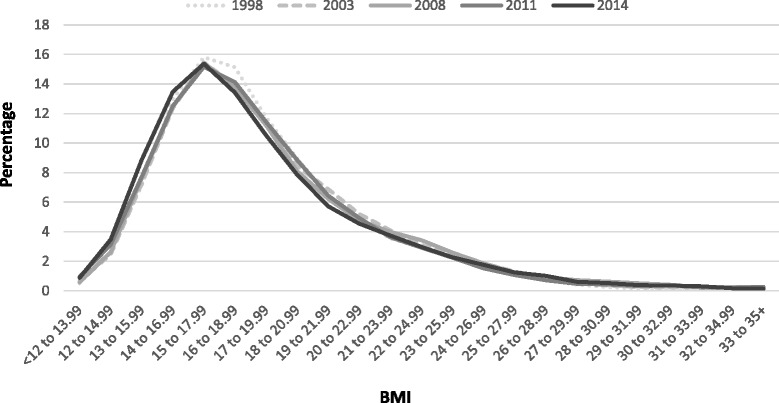
The percentage of children aged 2–15 years at each gradation of BMI from 1998 to 2014. Note: Selected years of data are shown to maintain reasonably consistent time intervals between *lines*. The BMI gradations on the x-axis overlap as they are 3-point moving averages

Figure 5 shows the trend in BMI distribution for children aged 2–15 years from 1998 to 2014 (with selected years’ data shown for ease of reading). The distribution of BMI remained fairly stable between 1998 and 2014. The age distribution for children in Scotland has changed marginally over time with fewer older children, and an increase in younger children since 1998, in the sample (and population), due to birth rate changes and migration patterns. Age-standardisation to account for this change over time did not impact the trend. The results from the quantile regression in Additional file 1: Table S2 and Additional file 4: Figure S2C suggest that the rate of change varied across the length of the distribution.

### Trends in BMI distribution by area deprivation

**Fig. 6:**
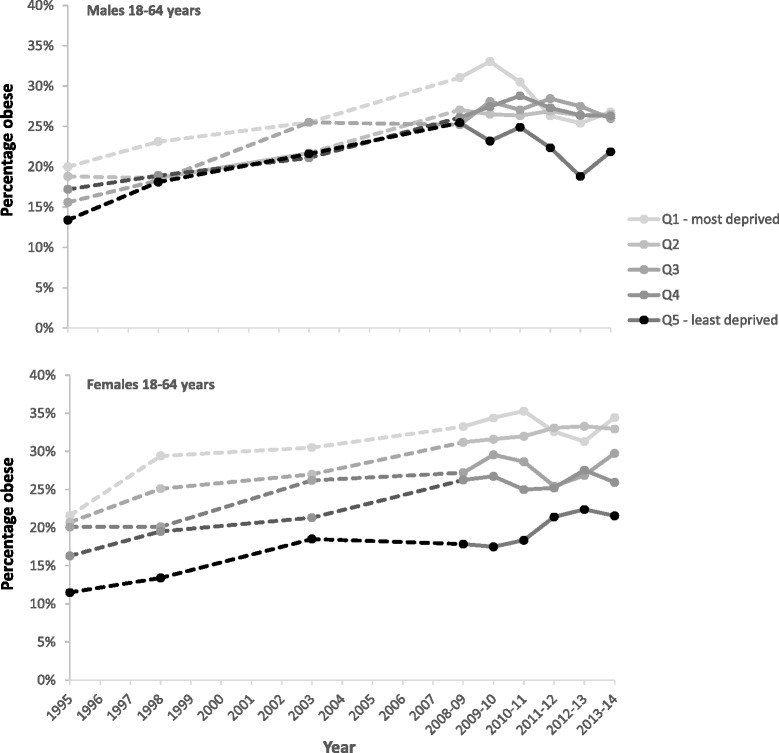
The percentage of men and women aged 18–64 years who are obese (BMI >30) by SIMD quintile (1995–2014). Note: the data from 2008 onwards use 2-year moving averages (2008–09 = average of 2008 & 2009; 2009–10 = average of 2009 & 2010)

Figure 6 shows the patterning of obesity prevalence in men and women by deprivation quintiles. In addition to highlighting inequalities in obesity prevalence between the most and least deprived areas, Fig. 6 also illustrates gradients in prevalence across the deprivation spectrum. Among men, obesity prevalence followed a gradient with SIMD quintile at the start of the time series (1995) with obesity increasing steadily as deprivation increased. However, while obesity has tended to be highest for those in the most deprived quintile, and lowest for those in the least deprived quintile across the series, the more recent years suggest that levels in the least deprived quintile have declined while the remaining four quintiles have converged (and stabilised). In contrast, a persistent gradient in obesity prevalence is evident among women for most of the 1995–2014 period, with levels progressively higher as deprivation increases. This gender-deprivation pattern in obesity levels is also evident in Additional file 5: Figure S3 which illustrates the relative index of inequality (RII) and slope index of inequality (SII) across SIMD quintiles for men and women.

**Fig. 7:**
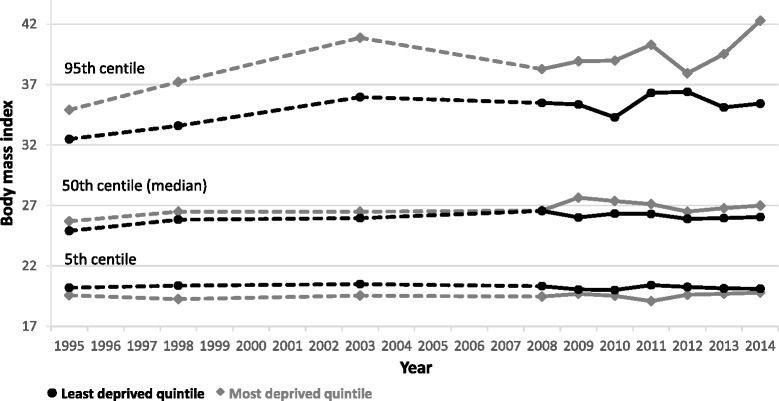
BMI values at the 5th, median and 95th centiles, from 1995 to 2014 stratified by SIMD quintile

Figure 7 illustrates how the BMI value centile distribution patterns seen so far differ by deprivation quintile. For ease of interpretation, only the most and least deprived quintiles are compared. BMI values have been consistently higher at the upper end of the distribution among adults aged 18–64 years living in the most deprived areas than is the case for those in the least deprived areas. As before, both groups have followed similar trajectories in terms of the overall increase in mean BMI. However, there is also some suggestion that the stabilising of trends seen in recent years for the whole population at the 95th centile has not occurred among those in the most deprived areas. However, more years’ data are needed to confirm this pattern (the 2014 peak could well be an outlier). In contrast, the median and 5th centile values have been consistently similar in the most and least deprived quintiles (and have followed the same patterns as seen in the whole population).

**Fig. 8:**
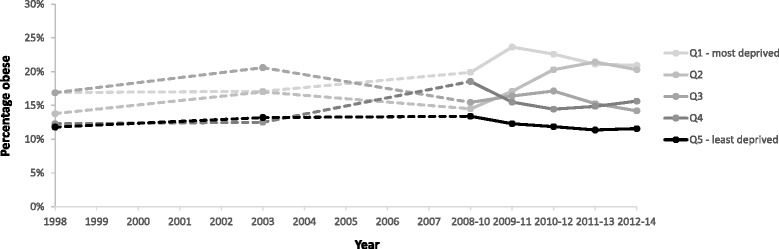
The percentage of children aged 2–15 years classified at risk of obesity (BMI >95th percentile) by SIMD (1998–2014). Note: the data from 2008 onwards use 3-year moving averages (2008–10 = average of 2008, 2009 & 2010; 2009–11 = average of 2009, 2010 & 2011)

**Fig. 9:**
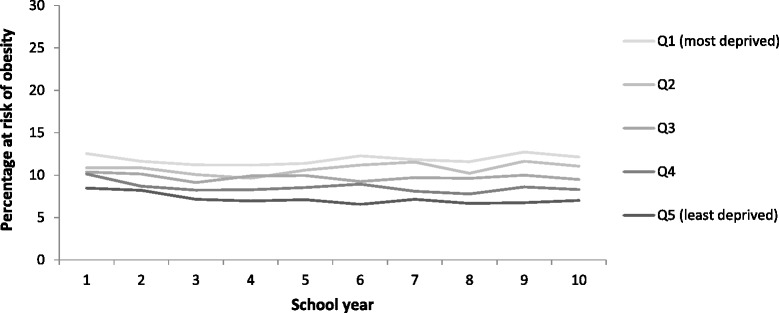
The percentage of P1 children classified at risk of obesity (BMI >95th percentile) by SIMD (2005/6 – 2014/15)

While the patterning of obesity prevalence by SIMD has changed over time for men and women, the size of the gap between the least and most deprived groups has not widened significantly. In contrast, Fig. 8 illustrates how inequalities in epidemiologically-defined obesity amongst children aged 2–15 years have widened. This is largely a consequence of obesity levels amongst the least deprived children remaining static while they increased for those in the two most deprived quintiles. This upward trend among children in the two most deprived quintiles was significant (p = 0.02) whereas the prevalence among children in the two least deprived quintiles did not vary significantly (p = 0.10). The data for P1 children from the CHSP show a persistent inequality in the prevalence of obesity over time (Fig. 9). It is less clear whether this gap has widened in recent years because of marked differences in local authority participation rates over time.

## Discussion

### Main findings of this study

The BMI distribution amongst adults shifted rightwards, with a large proportion of the population gaining a small amount of weight, between 1995 and 2008, since when the pattern has largely stabilised. This indicates both a whole population shift in the BMI distribution over time, and a disproportionate increase for those already with the highest BMIs. Analysis of population survey data collected in England between 1992 and 2013 showed similar upward trends in BMI values at the median and 95th centiles in adults aged 16 and over [5]. However, in contrast, the values at the 95th centile in England have continued to increase, whereas the overall values in Scotland stabilised at the same time that the median values for the pattern did (though they appear to have continued increasing among people living in the most deprived areas). Additional analysis of the Scottish trend for all adults aged 18 years and over from 2003 onwards showed a similarly flat line for the 95th centile values (Additional file 6: Figure S4), so the exclusion of adults aged 65 years and over from the Scottish data does not appear to have caused this divergence of patterns.

Inequalities in adult obesity prevalence have persisted over time, though with different underlying patterns evident for men and women. In contrast, while levels of child obesity have been broadly stable over time, inequalities in the prevalence of obesity have widened. This is largely due to obesity increasing among children from the two most deprived areas while remaining stable for those in the least deprived areas. Data on P1 children cautiously suggest a persistent gap over time, though the pattern is less conclusive.

### Strengths and limitations of this study

This study used the best available data monitoring trends in BMI and obesity in Scotland, including objectively collected data which did not rely on self-reporting. However, there are a number of important limitations in the data sources used in this study.

The sample sizes in some of the later years of the SHeS were relatively small, especially after stratification, and this created some year-to- year fluctuations in the trends that were likely to be due to random sampling variation. We overcame this using pooled data and rolling averages, where possible. For the P1 child data, the participation rate of local authorities increased markedly over time and there may have been systematic biases in the trends introduced by the greater participation of more deprived areas towards the end of the time series. Further work is required to understand the impact of the missing older data on obesity prevalence in P1 children. Given the differential trends in obesity prevalence across deprivation groups, the (lack of an) overall population trend for P1 children should not be over-interpreted. However, the trend among all children aged 2–15 years, monitored in the SHeS, shows the same pattern (of relative stability). Similarly, the response rate of adults in the height and weight data collection in the SHeS decreased from the earlier waves, and there may have been systematic biases introduced by this (though the extent of the decline is exaggerated due to the lack of age-specific response data). It seems likely that non-respondents to the survey are less likely to be healthy than respondents [15].

In 1995 and 1998 one adult per household was selected for the survey, from 2003 onwards all adults (to a maximum of 10) were selected. This has the advantage of removing the need for selection weights (which increase standard errors), however this design will have increased standard errors due to weight patterns clustering within households. We believe this is unlikely to have impacted on the conclusions reported here as the increasing levels of BMI both predate and continue after this change to the sample design. Furthermore, the majority of the analyses presented here are stratified by sex, which will have effectively de-clustered the sample in the majority of households [16].

The declining response rates would introduce bias if the BMI levels of non-responders differed systematically to those of responders. The impact of non-response bias on BMI estimates is difficult to assess because the BMI (or other related health traits) of non-responders is unknown. This is further compounded by the absence of other sources of population BMI against which to assess the survey’s estimates. It is known that non-responders as a whole tend to be less healthy than responders and this has obvious consequences for the estimates reported in health surveys. The profile of the sample used for the analysis presented here has seen a small but statistically significant decline over time in the proportion of male and female respondents drawn from the most deprived quintile. The fact that the proportion of participants from these groups has declined by a few percentage points for both men and women is unlikely to have caused the distinct and diverging obesity patterns reported here for men and women by SIMD. However, it is plausible that the level of obesity in the most deprived areas is underestimated using these data, and that obesity inequality by SIMD is therefore even greater in the whole population. The survey does, however, use non-response weighting to adjust the estimates, drawing on the age, sex and deprivation level of household members who do not take part (for households where at least one adult participated), while area-level characteristics are used in the non-response weighting for households where no-one participated.

The sampling frame for SHeS excludes institutionalised populations which could have biased the results towards healthier participants. However, the age stratification used (and restriction to those aged 18–64 years) is likely to have reduced much of the risk of bias resulting from this. However, the age stratification bands used were broad and there may have been some small remaining confounding due to changes in the age composition of the population over time.

We used the entire SIMD index rather than income domain for allocating populations to different deprivation strata. This carries a small risk of reverse causation (as the full SIMD index includes health outcomes). However, the income domain has been shown to be highly correlated to the SIMD overall therefore it is likely that the risk is minimal [17]. As SIMD is a measure of area-deprivation we cannot draw conclusions about individuals and the measure is therefore subject to ecological fallacy as not all people living in the most deprived areas will be deprived and vice-versa. It is however useful as it is a multi-faceted measure that includes a number of dimensions important for identifying deprivation [18].

As with all area-based measures of deprivation, it is possible compositional change in where people live rather than changes in outcomes for those populations is responsible for the observed trends in inequalities. Longitudinal studies which track individual movement between areas in different deprivation strata are not currently available in Scotland to assess the extent to which this might exacerbate or minimise (if there is a move towards homogeneity or heterogeneity in community composition) inequalities who are individually deprived. This limitation has to be balanced against the limitations of other measures of socio-economic position which often misclassify or exclude particular groups.

### How this fits with the existing literature

The causes of the rise in obesity are complex and involve changes across the food industry, trade, transport policy, work and the economy more broadly [1]. The Scottish Government has a strategy which details the challenge obesity presents in Scotland [19], though the actions progressed in the short-term focussed on individual level interventions requiring participants to “opt-in” [20] with less attention given to actions which address the economic and legislative aspects, especially on features of the obesogenic environment [21]. It is those aspects of policy that underpin population-wide interventions to reduce inequalities which are likely to be more effective as they do not rely on individual agency to achieve change [22]. More comprehensive actions in these areas may succeed in achieving both a population-wide decrease in obesity as well as a decrease in obesity inequalities.

The successful enactment of these policies relies on their collective agreement and implementation by relevant public agencies [23]. However, it may be that the recent stabilisation of the obesity trends can be explained by the economic downturn and the implications this has had for the affordability of food, rather than the result of policy change [24].

### Implications

Whole population shifts, even of quite small degrees for each individual, can result in large changes in the total proportion of a population at risk of harms [25]. Rose [21] estimated, for example, that an average decrease in weight of 1 kg across a whole population could yield a 25% reduction in obesity prevalence in that population [21]. The BMI distributions shown here are one such example of how a small overall shift in the distribution, or how a small change in the skew of the population, can lead to a substantial increase in the population who are obese.

Parallels with other risks to health, such as those associated with alcohol, can be clearly drawn. In Scotland, at least, the strategy pursued in recent years has explicitly acknowledged the need for policies that target the heaviest drinkers (with the highest risk of harm) in conjunction with whole population approaches to reduce overall consumption [26]. Underlining this approach is the principle that “highly targeted interventions for those at greatest risk are necessary but not sufficient” [p10] [27]. This multi-stranded approach has widespread support amongst the health policy community and follows that recommended by the WHO. As Rose argues: “The abolition of deviance, whilst leaving the population as a whole unchanged, seems not to occur” [p103] [25]. Adopting a similar approach for obesity, that simultaneously targets both the heaviest people and the population as a whole, is likely to be the most effective way to reduce obesity-related harm and reverse the trends observed in this paper. However, measures to enhance access to the population-wide approach for those most badly affected or at greatest risk (‘proportionate universalism’) [28] would likely be necessary to help reduce inequalities. In addition, it is essential for policies to be sensitive to the differential experiences of obesity revealed in these analyses, such as the persistence of deprivation gradients among women and the convergence of patterns among men outside of the least deprived areas, and the widening of inequalities among children.

## Conclusions

The BMI distribution has seen an overall population shift to the right over time with a large proportion of the population gaining a small amount of weight. While this has stabilised in recent years, there are, as yet, no signs that it is reversing for the population as a whole, while there is a suggestion that BMI may have continued to increase among people at the highest end of the BMI distribution living in the most deprived areas. Given the population-wide impact of the trends, and the tentative rise in inequalities among children, actions to reduce the obesogenic environment are urgently needed if the long-term health, social and inequality consequences of obesity are to be reduced.

## Additional files

Additional file 1: Table S1. Quantile regression results for the 10th, 25th, 50th, 75th and 90th percentile year coefficients, by sex and broad age group. **Table S2.** Quantile regression results for the 10th, 25th, 50th, 75th and 90th percentile year coefficients, children aged 2 to 15 years. (PDF 196 kb)

Additional file 2: Figure S2A. Mean change in adult BMI from 1995 to 2014 against the rate of change in the 10th, 25th, 50th, 75th and 90th quantiles. (PDF 168 kb)

Additional file 3: Figure S2B. Mean change in BMI from 1995 to 2014 against the rate of change in the 10th, 25th, 50th, 75th and 90th quantiles, by sex and age group. (PDF 274 kb)

Additional file 4: Figure S2C. Mean change in BMI from 1998 to 2014 against the rate of change in the 10th, 25th, 50th, 75th and 90th quantiles, children aged 2–15 years. (PDF 169 kb)

Additional file 5: Figure S3. Relative Index of Inequality and Slope Index of Inequality for prevalence of obesity by SIMD quintile. (PDF 186 kb)

Additional file 6: Figure S4. BMI values at the 5th, median and 95th centiles from 2003 to 2014, all adults 18 years plus. (PDF 184 kb)

Additional file 7: Figure S1. The percentage of adults at each gradation of BMI for selected years between 1995 to 2014 (with three-point rolling means applied to provide smoother distributions). (PDF 201 kb)

## Abbreviations

BMI: Body Mass index
CHSP: Child Health Systems Programme
P1: Primary One
SHeS: Scottish Health Survey
SIMD: Scottish Index of Multiple Deprivation
WHO: World Health Organization
