# Supplementary material for: Obesity in Scotland: a persistent inequality
Source: Int J Equity Health. 2017 Jul 27;16:135. doi: 10.1186/s12939-017-0599-6 (PMC5530512; doi:10.1186/s12939-017-0599-6)
Supplement: Supplementary file 1 — Quantile regression results for the 10th, 25th, 50th, 75th and 90th percentile year coefficients, by sex and broad age group. Table S2. Quantile regression results for the 10th, 25th, 50th, 75th and 90th percentile year coefficients, children aged 2 to 15 years. (PDF 196 kb) [file 12939_2017_599_MOESM1_ESM.pdf]

Table S1 Quantile regression results for the 10th, 25th, 50th, 75th and 90th percentile year coefficients, by sex and broad age group

	OLS regression (mean)		10th quantile		25th quantile		50th quantile		75th quantile		90th quantile	
**Year effect**	**coefficient**	**95% CI**	**coefficient**	**95% CI**	**coefficient**	**95% CI**	**coefficient**	**95% CI**	**coefficient**	**95% CI**	**coefficient**	**95% CI**
**Adults**	0.170	0.153 to 0.187	0.054	0.041 to 0.067	0.086	0.072 to 0.101	0.148	0.130 to 0.166	0.239	0.212 to 0.267	0.314	0.271 to 0.357
**Males**												
18-44	0.131	0.102 to 0.160	0.046	0.014 to 0.078	0.077	0.046 to 0.109	0.092†	0.056 to 0.128	0.162†	0.111 to 0.214	0.265†	0.182 to 0.348
45-64	0.167	0.134 to 0.199	0.096	0.055 to 0.136	0.107	0.073 to 0.141	0.159†	0.127 to 0.191	0.206†	0.154 to 0.258	0.254†	0.167 to 0.342
**Females**												
18-44	0.240	0.206 to 0.273	0.047	0.022 to 0.071	0.104†	0.075 to 0.133	0.214†	0.171 to 0.257	0.387†	0.317 to 0.456	0.450†	0.337 to 0.562
45-64	0.131	0.094 to 0.168	0.035	-0.005 to 0.074	0.051	0.013 to 0.089	0.107†	0.073 to 0.142	0.209†	0.153 to 0.264	0.288†	0.206 to 0.370

* Adjusted for survey wave, single year of age (within age group) and SIMD
* Confidence intervals based on boot-strapped estimates (100 repetitions)
* Calculated on unweighted data (software restrictions)
† Coefficient statistically significantly higher than the coefficient for the 10th quantile ($p < 0.05$)

Table S2 Quantile regression results for the 10th, 25th, 50th, 75th and 90th percentile year coefficients, children aged 2 to 15 years

	OLS regression (mean)		10th quantile		25th quantile		50th quantile		75th quantile		90th quantile	
**Year effect**	**coefficient**	**95% CI**	**coefficient**	**95% CI**	**coefficient**	**95% CI**	**coefficient**	**95% CI**	**coefficient**	**95% CI**	**coefficient**	**95% CI**
2-15 years	-0.005	-0.024 to 0.015	-0.030	-0.047 to -0.014	-0.025	-0.043 to -0.007	-0.020	-0.038 to -0.002	0.006†	-0.024 to 0.036†	0.039†	-0.020 to 0.079†

* Adjusted for survey wave, single year of age (within age group), sex and SIMD
* Confidence intervals based on boot-strapped estimates (100 repetitions)
* Calculated on unweighted data (software restrictions)
† Coefficient statistically significantly higher than the coefficient for the 10th quantile ($p < 0.05$)
