# Supplementary material for: Obesity in Scotland: a persistent inequality
Source: Int J Equity Health. 2017 Jul 27;16:135. doi: 10.1186/s12939-017-0599-6 (PMC5530512; doi:10.1186/s12939-017-0599-6)

Figure S2A Mean change in adult BMI from 1995 to 2014 against the rate of change in the 10th, 25th, 50th, 75th and 90th quantiles

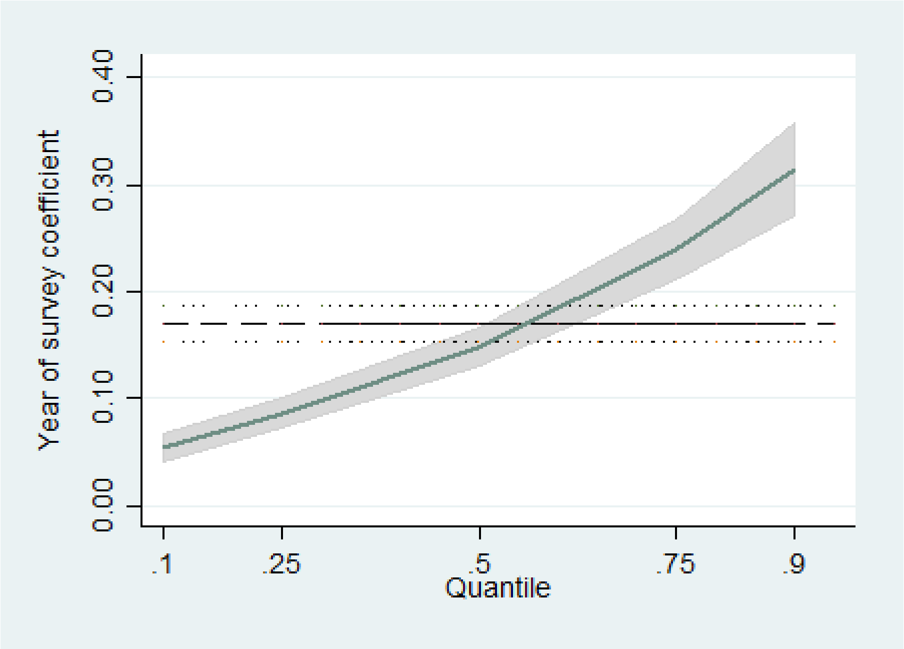

Supplement: Supplementary file 2 — Mean change in adult BMI from 1995 to 2014 against the rate of change in the 10th, 25th, 50th, 75th and 90th quantiles. (PDF 168 kb) [file 12939_2017_599_MOESM2_ESM.pdf]
