# Supplementary material for: Obesity in Scotland: a persistent inequality
Source: Int J Equity Health. 2017 Jul 27;16:135. doi: 10.1186/s12939-017-0599-6 (PMC5530512; doi:10.1186/s12939-017-0599-6)

Figure S2C Mean change in BMI from 1998 to 2014 against the rate of change in the 10th, 25th, 50th, 75th and 90th quantiles, children aged 2-15 years

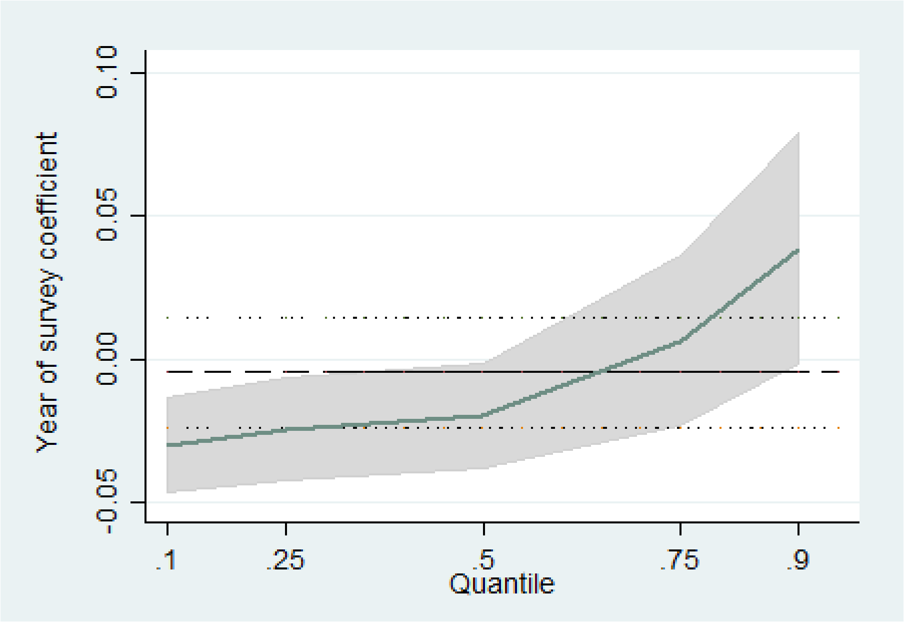

Supplement: Supplementary file 4 — Mean change in BMI from 1998 to 2014 against the rate of change in the 10th, 25th, 50th, 75th and 90th quantiles, children aged 2–15 years. (PDF 169 kb) [file 12939_2017_599_MOESM4_ESM.pdf]
