# Supplementary material for: Obesity in Scotland: a persistent inequality
Source: Int J Equity Health. 2017 Jul 27;16:135. doi: 10.1186/s12939-017-0599-6 (PMC5530512; doi:10.1186/s12939-017-0599-6)

Figure S3 Relative Index of Inequality and Slope Index of Inequality for prevalence of obesity by SIMD quintile

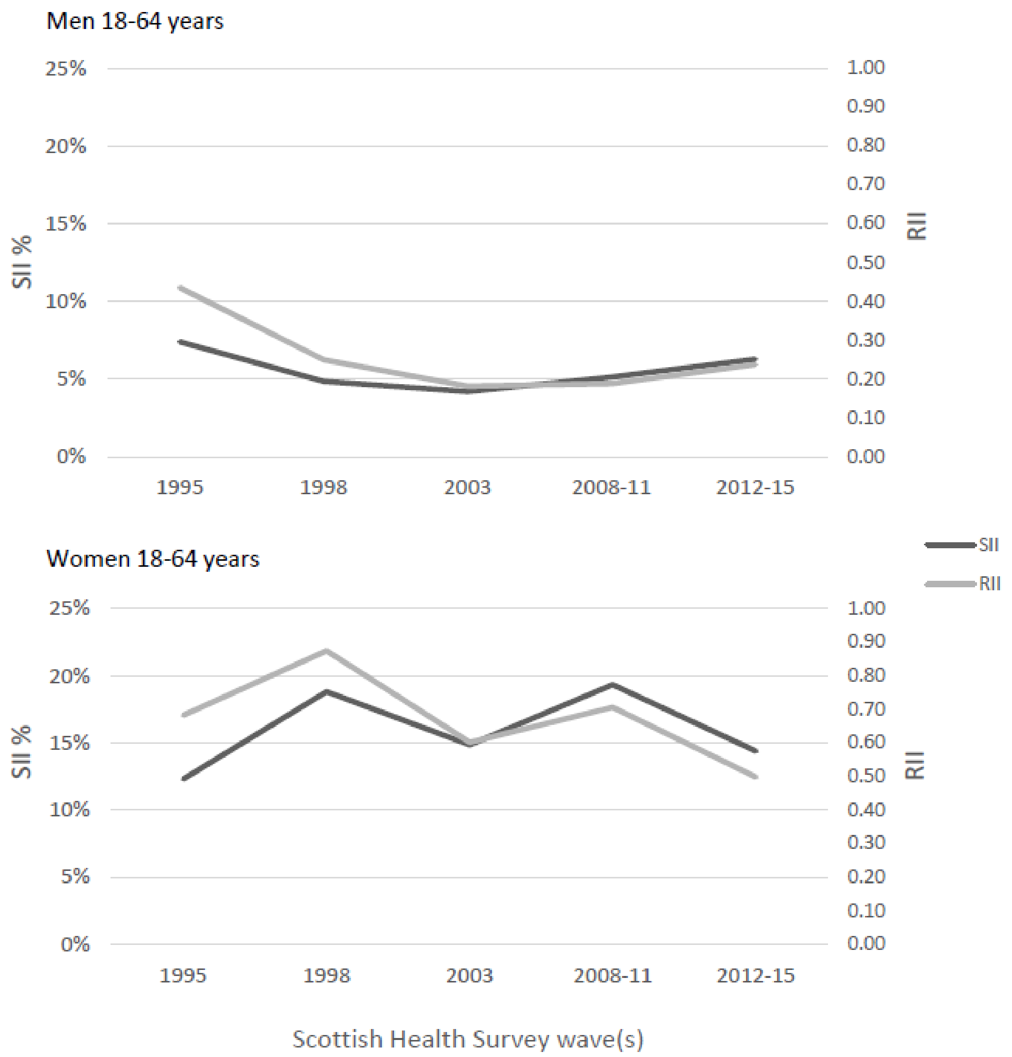

Supplement: Supplementary file 5 — Relative Index of Inequality and Slope Index of Inequality for prevalence of obesity by SIMD quintile. (PDF 186 kb) [file 12939_2017_599_MOESM5_ESM.pdf]
