# Supplementary figures and images for: Obesity in Scotland: a persistent inequality
Source: Int J Equity Health. 2017 Jul 27;16:135. doi: 10.1186/s12939-017-0599-6 (PMC5530512; doi:10.1186/s12939-017-0599-6)

**Figure S4** BMI values at the 5<sup>th</sup>, median and 95<sup>th</sup> centiles from 2003 to 2014, all adults 18 years plus

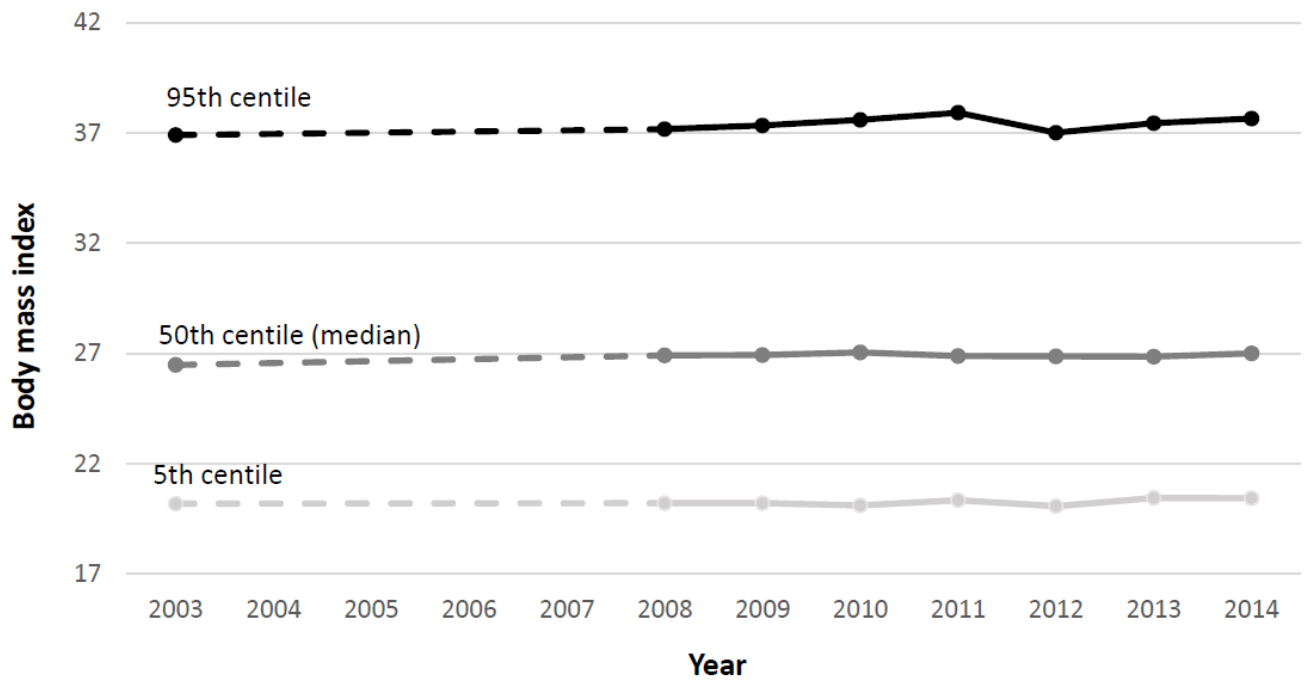

Supplement: Supplementary file 6 — BMI values at the 5th, median and 95th centiles from 2003 to 2014, all adults 18 years plus. (PDF 184 kb) [file 12939_2017_599_MOESM6_ESM.pdf]
