# Supplementary material for: Obesity in Scotland: a persistent inequality
Source: Int J Equity Health. 2017 Jul 27;16:135. doi: 10.1186/s12939-017-0599-6 (PMC5530512; doi:10.1186/s12939-017-0599-6)

Figure S1 The percentage of adults, aged 18-64 years, at each gradation of BMI for selected years between 1995 to 2014 (with three-point rolling means applied to provide smoother distributions)

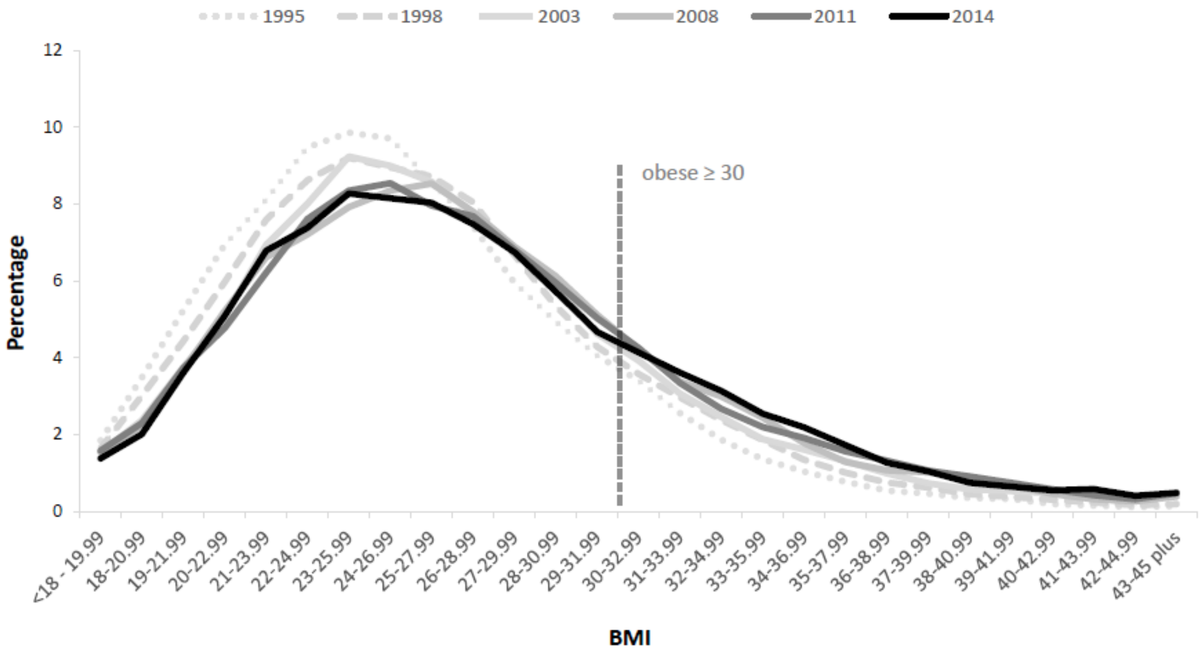

Supplement: Supplementary file 7 — The percentage of adults at each gradation of BMI for selected years between 1995 to 2014 (with three-point rolling means applied to provide smoother distributions). (PDF 201 kb) [file 12939_2017_599_MOESM7_ESM.pdf]
